# Interconnected environmental ethics: navigating human, animal, and planetary health in the climate crisis

**DOI:** 10.1080/11287462.2025.2483053

**Published:** 2025-04-07

**Authors:** Elizabeth Cerceo

**Affiliations:** Department of Medicine, Cooper Medical School of Rowan University, Cooper University Health Care, Camden, NJ, USA

## The climate crisis: a direct threat to human and animal health

The World Health Organization has identified climate change, driven by global reliance on fossil fuels, as the greatest threat to global health in the 21st century. Physicians are already treating the acute and chronic health effects from climate change, but they are treated as disparate events, sending our patients back to the larger context driving their illnesses: from wildfire smoke triggering emphysema exacerbations, to heatwaves contributing to myocardial infarctions, or particulate matter pollution worsening asthma control. Extreme weather events exacerbate chronic conditions and mental health crises. Waterborne infections (leptospirosis, cryptosporidiosis, vibrio infections) and vector-borne illnesses, like malaria, Lyme disease, and dengue fever, are expanding as ecosystems shift and extreme storms worsen.

However, climate change also affects animal health, creating rippling impacts that rebound back to humans. Intense drought has precipitated declines in cattle herd size, down to a historic low, impacting ranching and farming. While commodification of livestock animals places threats as a potential economic loss, it denies the inherent worth of other living creatures and downplays the impacts of climate wrought on vulnerable populations (U.S. Agricultural Commodities in Drought, 2025). In the midst of extreme heat in 2023, reports of howler monkeys falling dead out of trees abounded across Mexico (Howler Monkeys Are Falling From Trees Amid Mexico’s Brutal Heat Wave, 2025). The endangered giant kangaroo rat, which is native to dry habitats in California, has adapted to occasional short droughts but multi-year droughts are causing existential threats.

Zoonotic diseases, infections that jump from animals to humans, account for over 60% of emerging infectious diseases globally, and their frequency is increasing in the context of climate change. As rising temperatures, deforestation, and habitat loss disrupt ecosystems, animals are forced into closer contact with humans, creating new opportunities for disease spillover. Changes in climate patterns, such as altered rainfall and warming, also expand the geographic range of disease vectors like mosquitoes and ticks, spreading illnesses such as malaria, dengue, and Lyme disease. The COVID-19 pandemic is a stark reminder of how interconnected human, animal, and environmental health truly are. While its exact origins remain under investigation, COVID-19 highlights the risks of zoonotic spillovers in a world where human activity accelerates environmental degradation (Gibb et al., 2025; Kuhn et al., 2024; Marchi et al., 2024). Changes in temperature and precipitation allow disease vectors, such as mosquitoes and ticks, to thrive in new regions, infecting both humans and animals. Upticks in West Nile virus and Lyme infections illustrate the increasing overlap between human and animal disease burdens.

The One Health perspective is critical to addressing zoonotic diseases like COVID-19, as it emphasizes the interconnected nature of health across species and ecosystems (Gibb et al., 2025; Kuhn et al., 2024; Marchi et al., 2024). It also offers a lens through which to view climate and environmental impacts. Climate change and human encroachment into wildlife habitats not only harm biodiversity but also increase the likelihood of future pandemics by disrupting natural disease regulation. Preventing such crises requires interdisciplinary collaboration between physicians, veterinarians, environmental scientists, and policymakers. By prioritizing climate action, wildlife conservation, and improved surveillance of zoonotic threats, we can mitigate the drivers of disease emergence. Adopting a One Health approach ensures we focus not only on human health but also on the broader systems that sustain it – recognizing that a healthy planet is the foundation for healthy people and animals alike.

## Interdependence of the human-animal dyad

Beyond direct health impacts, climate change and ecological disruption also give rise to intricate, cascading interactions within ecosystems that can have surprising and serious consequences for human health—effects that require a more nuanced, systems-level approach to fully understand and address. For example, when bat populations were decimated by white-nose disease, human infant mortality rates rose by ∼8%, correlating to 1,334 infant deaths between 2006–2017 (Frank, 2024). This occurs when bat colonies are infected with the fungus *Pseudogymnoascus destructans,* leading to plummeting bat populations which then results in a rising insect burden (White-Nose Syndrome, 2025). A single bat consumes up to 40% of its body weight in insects every night. In counties with outbreaks of white-nose syndrome, farmers used 31% more pesticides which was linked to the increased infant deaths, even after controlling or the opioid epidemic, parental unemployment, other weather factors, and genetically modified organisms.

These “ecosystem services” are not limited to effects on insect populations (Ecosystem Services – USDA Climate Hubs, 2025). When vultures died off in large numbers in India after cattle were given the anti-inflammatory diclofenac for arthritis, nearly half a million people died from 2000–2005 from affects on water quality and increases in rabies (NY Times - How a crisis for vultures led to a human disaster, 2025). Governments in Europe and South Asia are still not regulating veterinary drugs sufficiently to protect vultures (Cook et al., 2024). There is growing public awareness and concern surrounding the widespread use—and often overuse—of antibiotics in livestock, which has been increasingly linked to the emergence and spread of antimicrobial resistance, a pressing global health threat.

Loss of ash trees to the invasive emerald ash borer increased deaths related to cardiovascular and respiratory illness, through stress, decreased physical activity, and poorer air quality (Donovan et al., 2013). Reintroduction of wolves to Wisconsin reduced car crashes involving deer by 24% with most of the reduction due to a behavioral response of deer to wolves rather than through a deer population decline from wolf predation, limiting overabundant deer in ways that human deer hunters cannot (Raynor et al., 2021). This benefit to human health led to an economic benefit that is 63 times greater than the costs of predation on livestock. The loss of such ecosystem services highlights the potential devastating impact of the extinction of species or even when local systems are only diminished, which has been accelerating in recent years (Chivian & Bernstein, 2008; Halley & Pimm, 2023; Metzen & Ocklenburg, 2024; One million species are on the brink of extinction, 2025; Raven, 2020; Shivanna, 2020).

## The ethical responsibility to act: prevention and mitigation

Physicians hold a unique position of trust in society (Campbell et al., 2023). That trust, based in our ethical mandate, requires us to advocate for climate action that prevents further harm to the health of humans, animals, and the planet. This ethical duty aligns with the preventive nature of medicine: rather than treating disease reactively, we must proactively mitigate the root causes of the climate crisis and ecosystem degradation. Just as we counsel patients to reduce risk factors like smoking or sedentary behavior, we must speak out against systemic threats like fossil fuel dependence and environmental destruction. In doing so, we extend the practice of medicine beyond the clinic walls and into the broader determinants of health that shape the well-being of current and future generations of humans and other species.

## Areas where physicians can lead

*Advocating for Climate-Resilient Policies and Systems*: Physicians must advocate for policies that reduce greenhouse gas emissions, promote renewable energy, and protect ecosystems, all of which prevent harm to health across species. Whether at the local, state, or federal level, reducing air pollution through cleaner energy policies not only mitigates climate change but also reduces rates of asthma and cardiovascular disease in humans and animals (Jiang et al., 2024; Rajagopalan & Landrigan, 2021; Sagheer et al., 2023). Protecting ecosystems through land conservation and promotion of indigenous practices safeguards biodiversity, reducing the risk of zoonotic spillover events (Clifford Astbury et al., 2023; Glidden et al., 2021). Supporting climate-smart food systems and prioritizing more healthful plant-based diets benefit public health while improving the welfare of livestock and wildlife (Cerceo et al., 2024; Kalwaney & Cerceo, 2024).

*Integrating One Health into Clinical Practice and Education*: Medical education and practice must integrate climate literacy and the principles of One Health (Mackenzie & Jeggo, 2019). Understanding the intersection between environmental changes, animal health, and human health prepares physicians to anticipate climate-related health challenges, such as emerging zoonotic diseases or food insecurity. Programs that teach future physicians about the connections between climate and health enable us to better diagnose, treat, and advocate for vulnerable patients and communities.

*Reducing Healthcare’s Environmental Footprint*: The healthcare sector itself contributes ∼8.5% of greenhouse gas emissions in the United States (Eckelman et al., 2020; Thiel & Richie, 2022). Physicians must support efforts to transition to sustainable healthcare practices, including promoting energy efficiency in hospitals and clinics, reducing medical waste and unnecessary single-use plastics, eliminating desflurane and other anesthetics many times more potent than carbon dioxide in the environment, and advocating for greener supply chains. These actions reflect a commitment to do no harm – to patients, to the planet, and to the interconnected web of life.

The climate crisis demands a shift in how we view health: as something deeply intertwined with the well-being of animals and ecosystems. Physicians cannot view their ethical responsibility as ending at the bedside. Instead, we must expand our understanding of health to include environmental stewardship and interdisciplinary collaboration. The One Health framework provides a path forward – one that aligns with our professional duty to heal and prevent harm. By addressing the root causes of the climate crisis, advocating for policies that promote environmental and animal health, and transforming healthcare systems to be more sustainable, physicians can become ethical leaders in this fight.

## Conclusion

As the impacts of the climate crisis accelerate, the principle of do no harm must guide us toward bold action. Physicians have a moral and professional responsibility to lead the charge for climate solutions that protect humans, animals, and ecosystems. The One Health approach acknowledges this interconnectedness, advocating for interdisciplinary collaboration between human health professionals, veterinarians, environmental scientists, and policymakers. By addressing these connections, physicians can help advocate for healthy systems that protect all life, recognizing that harm to animals and ecosystems ultimately harms human health.

“The health of humans, animals, and the environment is inseparable. To care for one, we must care for all.”
